# ROS induced pyroptosis in inflammatory disease and cancer

**DOI:** 10.3389/fimmu.2024.1378990

**Published:** 2024-07-01

**Authors:** Jingsong Wang, Ziyong Wu, Min Zhu, Yang Zhao, Jingwen Xie

**Affiliations:** ^1^ Department of Pharmacy, Guangyuan Central Hospital, Guangyuan, Sichuan, China; ^2^ Department of Pharmacy, Ezhou Central Hospital, Ezhou, Hubei, China; ^3^ Department of Pharmacy, Xuchang Central Hospital, Xuchang, Henan, China; ^4^ Department of Health, Chongqing Industry & Trade Polytechnic, Chongqing, China

**Keywords:** ROS, pyroptosis, inflammation, tumor, cell death

## Abstract

Pyroptosis, a form of caspase-1-dependent cell death, also known as inflammation-dependent death, plays a crucial role in diseases such as stroke, heart disease, or tumors. Since its elucidation, pyroptosis has attracted widespread attention from various sectors. Reactive oxygen species (ROS) can regulate numerous cellular signaling pathways. Through further research on ROS and pyroptosis, the level of ROS has been revealed to be pivotal for the occurrence of pyroptosis, establishing a close relationship between the two. This review primarily focuses on the molecular mechanisms of ROS and pyroptosis in tumors and inflammatory diseases, exploring key proteins that may serve as drug targets linking ROS and pyroptosis and emerging fields targeting pyroptosis. Additionally, the potential future development of compounds and proteins that influence ROS-regulated cell pyroptosis is anticipated, aiming to provide insights for the development of anti-tumor and anti-inflammatory drugs.

## 1 Introduction

Reactive oxygen species (ROS) encompass a collective term for oxygen-containing and highly reactive substances present in organisms or the natural environment. These substances include superoxide anion (O^2−^), hydrogen peroxide (H_2_O_2_), nitric oxide (NO), and peroxyl radical (OH•) (1–4). These oxygenated compounds are involved in numerous physiological activities that influence the state of human health and stabilize the immune system (5–7). Generally, a moderate level of ROS promotes cell growth, reproduction, and repair, but excessive ROS could aggravate disease symptoms and impede wound healing (8–10). Moreover, the overexpression of ROS plays a key role in cell death, including ferroptosis, apoptosis, and necrosis (11). They are also considered significant factors in many cellular signaling events, with excessive levels of ROS reported in various diseases being responsible for promoting pyroptosis (12, 13).

Pyroptosis, an untypical form of programmed cell death, was discovered in recent years (14). Unlike other forms of cell death, pyroptosis is characterized by the formation of pores on the cell membrane, loss of integrity, fluid influx, cell swelling, leakage of cellular contents, increased permeability, cell membrane rupture, and induction of inflammatory reactions (15–17). Pyroptosis is closely related to the occurrence of certain diseases, such as cancer, infectious diseases, cardiovascular diseases, and central nervous system diseases (18). Originally, pyroptosis was reported as a caspase-1-dependent cell death (19). The primary reason is the NOD-like receptor thermal protein domain associated protein 3 (NLRP3) inflammasome is primed by nuclear factor kappa-B (NF-κB), resulting in the activation of caspase-1. Caspase-1 then cleaves GSDMD, leading to the oligomerization of GSDMD-NT and the formation of pores, subsequently inducing pyroptosis (20). However, other inflammatory caspases (caspase-4/-5/-11) and apoptosis-related caspases (caspase-3/-7/-8) have been found to also participate in pyroptosis (21–23). Substantial evidence indicates that ROS is an important factor causing pyroptosis, and high levels of intracellular ROS can induce pyroptosis in inflammatory or cancer cells (24, 25). Research has elucidated the role and mechanisms of pyroptosis in both tumors and inflammation, categorizing it into classical and non-classical pathways, among others (26). However, there is still a lack of a systematic summary of the role and mechanism of ROS in the development and progression of pyroptosis.

Here, we aim to elucidate the specific relationship between ROS and pyroptosis in diverse diseases, including inflammatory diseases and tumors, by combining multiple current research findings. Additionally, we intend to investigate the molecular mechanisms related to ROS and pyroptosis in multiple diseases and seek potential beneficial drugs for human health, providing novel therapeutic targets for ROS-induced pyroptosis-related diseases.

## 2 Caspase and gasdermin families in pyroptosis

### 2.1 Role of the caspase families in pyroptosis

Eukaryotic cells can initiate several self-destruction programs, with caspase-1-dependent cell death (known as pyroptosis) representing an intrinsic inflammatory response triggered by various pathological stimuli, such as stroke, heart disease, or cancer (27). Caspase-1-induced pyroptosis can occur independently of interleukin-1β (IL-1β) and interleukin-18 (IL-18) (28, 29). In the absence of IL-1β, Nucleotide-binding leucine-rich repeat-containing receptor 1a (NLRP1a) inflammasomes in hematopoietic progenitor cells can activate pyroptosis, acting as a cellular sentinel to alert caspase-1 (30). Additionally, the activation of the inflammatory sensor NLRP1b can activate pro-caspase-1-mediated cell death (31). After HIV infection, 95% of CD4 T cells undergo caspase-1-induced pyroptosis, with caspase-3-mediated apoptosis accounting for only a small fraction (32).

The mechanisms triggering inflammatory caspases to induce pyroptotic cell death are mostly unexplained. Researchers have observed that caspase-11 cleaves the transmembrane channel receptor-dependent pyroptosis (33). Dahai Yang et al. found that cytosolic lipopolysaccharide (LPS) stimulation induced caspase-11-dependent cleavage of the pannexin-1 channel and ATP release. This, in turn, activated the purinergic P2X7 receptor to mediate cytotoxicity, highlighting the critical roles of pannexin-1 and P2X7 in pyroptosis (34). However, bacterial virulence mechanisms, such as ADP-ribosylation of arginine, can inhibit caspase-11-mediated pyroptosis (35).

Caspase-8 serves as the initiator caspase of extrinsic apoptosis and represents the molecular switch controlling apoptosis, necroptosis, and pyroptosis, preventing tissue damage during embryonic development and adulthood (36). In macrophages, the inhibition of transforming growth factor-β-activated kinase 1 (TAK1) by pathogenic Yersinia triggers receptor-interacting serine/threonine-protein kinase 1 (RIPK1), caspase-8-dependent cleavage of gasdermin D (GSDMD), and inflammatory cell death (pyroptosis) (37). Moreover, programmed cell death-ligand 1 (PD-L1) converts tumor necrosis factor-alpha (TNF-α)-induced cell death into pyroptosis in cancer cells, resulting in tumor necrosis, primarily dependent on the noncanonical pyroptosis pathway mediated by GSDMC/caspase-8 (38). The cleavage of the caspase substrate GSDMD is sufficient to induce pyroptosis (39), and evidence suggests that cleavage of the substrate GSDMD by inflammatory caspases is ultimately required for pyroptosis (40).

### 2.2 Role of the gasdermin families in pyroptosis

GSDMD is another crucial component of the inflammasome, present in the NLRP3 inflammasome and serving as a substrate of caspase-1. GSDMD is required for pyroptosis (41). Pyroptosis involves membrane blebbing and the production of apoptotic-like cell protrusions (termed pyroptotic bodies) before the plasma membrane ruptures. GSDMD mediates pyroptosis after being cleaved by caspase-1 or caspase-11. The N-terminal fragment of GSDMD (GSDMD-NT) generated by caspase cleavage forms oligomers that migrate to the plasma membrane to induce pyroptosis by forming membrane pores (42, 43). The P10-form autoprocessed caspase-4/11 binds to the GSDMD-C domain with high affinity, and the Ragulator-Rag complex is essential for GSDMD pore formation and pyroptosis (12, 44). In addition to GSDMD cleavage, palmitoylation of GSDMD-NT, promoted by ROS and activated by inflammasomes, is a critical regulatory mechanism for controlling GSDMD membrane localization and activation (45), and reversible palmitoylation is a checkpoint for pore formation by both GSDMD-NT and intact GSDMD that serves as a general switch for the activation of this pore-forming family (46). However, GSDMD-NT, upon binding to phosphatidylinositol phosphates and phosphatidylserine (restricted to the inner leaflet of cell membranes) and cardiolipin (present in the inner and outer leaflets of bacterial membranes), undergoes mutations in four evolutionarily conserved basic residues that inhibit GSDMD-NT oligomerization, membrane binding, pore formation, and pyroptosis (47). GSDMD succination also hinders its interaction with caspases, limiting its processing, oligomerization, and induction of cell death (48).

**Figure 1:**
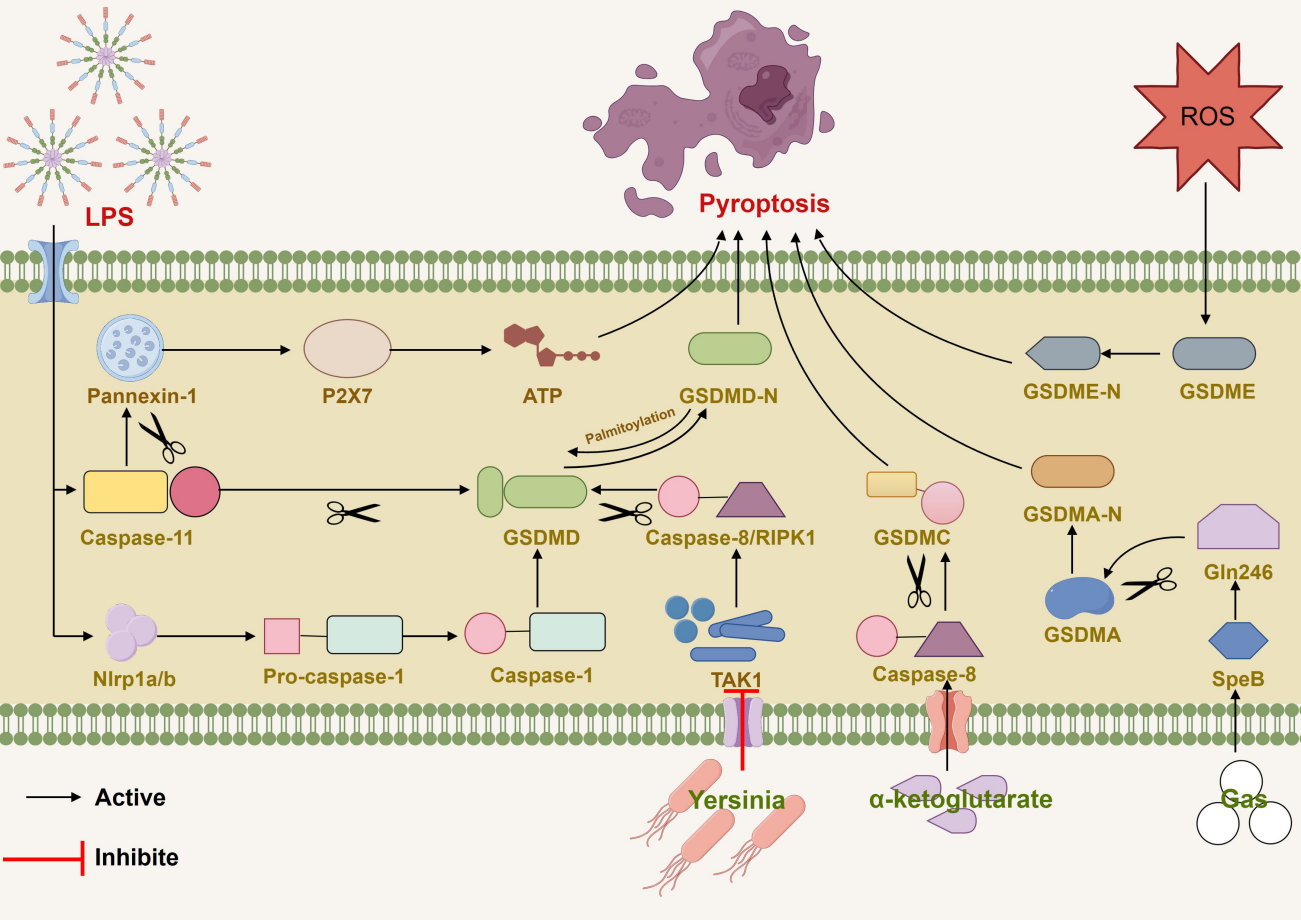
Various pathways induced pyroptosis by mediating caspase and gasdermin families (1). LPS induced caspase-11-dependent cleavage of the pannexin-1 channel and ATP release. (2) LPS stimulated NLRP1b and activate pro-caspase-1-mediated cell death. (3) ROS induced GSDME-dependent pyroptosis. (4) Inhibition of TAK1 by pathogenic Yersinia triggered RIPK1or caspase-8-dependent cleavage of GSDMD. (5) α-ketoglutarate induced pyroptosis through caspase-8-mediated cleavage of GSDMC. (6) GAS actived SpeB and triggered the release of active N-terminal fragments of keratinocytes through cleaving GSDMA after Gln246.

Gasdermin E (GSDME), originally identified as deafness, autosomal dominant 5 (DFNA5), is cleaved specifically by caspase-3 in the linker, generating a GSDME-N fragment that penetrates the membrane, thereby inducing pyroptosis (21). Activated ROS and released IL-1α can induce GSDME-dependent pyroptosis (49, 50). Gasdermin B (GSDMB) is the least studied among gasdermin family members, but research suggests that GSDMB is a critical factor in restoring epithelial barrier function and resolving inflammation (51), and interferon-γ (IFN-γ) upregulates GSDMB and promotes pyroptosis (52). Additionally, the metabolite α-ketoglutarate (α-KG) induces pyroptosis through caspase-8-mediated cleavage of gasdermin C (GSDMC) (53). Wanyan Deng et al. demonstrated that in Streptococcus pyogenes, also known as group A Streptococcus (GAS), the cysteine protease streptococcal pyrogenic exotoxin B (SpeB) virulence factor triggers the release of active N-terminal fragments of keratinocytes through cleaving gasdermin A (GSDMA) after Gln246, initiating pyroptosis (54). GSDMA plays a crucial role in the immune defense against invasive GAS skin infections and functions similarly to protective proteins (55). **Figure 1**

## 3 ROS regulates pyroptosis in inflammations

### 3.1 The negative effect of ROS regulates pyroptosis in inflammations

Recent evidence suggests that ROS plays a crucial role in inducing the NLRP3 inflammasome and releasing pro-inflammatory factors such as IL-3 and TNF in multiple inflammatory disorders (56). Current studies have indicated that elevated levels of ROS can activate NLRP3 and induce cellular pyroptosis (57), whereas blocking ROS production can inhibit NLRP3-dependent pyroptosis (58). This suggests a close correlation between the levels of ROS and the activation of NLRP3 inflammasomes and affect pyroptosis through NLRP3. Emerging evidence reports that MCC950, a specific NLRP3 inhibitor, reduces cigarette smoke extract (CSE)-induced pyroptosis (59, 60). Pretreatment with N-acetylcysteine (NAC), a ROS scavenger, has been indicated to inhibit CSE-induced pyroptosis (60). Meanwhile, NAC protects normal human intestinal epithelial cells (HIEC-6) from LPS-induced injury by reversing the activation of NLRP3 inflammasome-mediated pyroptosis (61). These pieces of evidence further demonstrate a positive association between ROS levels and NLRP3 activation, with NLRP3 serving as an indispensable key node in the process of ROS-mediated pyroptosis. Furthermore, the ROS/NLRP3 axis has been identified in hepatocytes, vascular endothelial cells, cardiomyocytes, and other cell types, regulating pyroptosis dependent on inflammatory factors (58, 62, 63). In summary, the overexpression of ROS can induce the expression of NLRP3, triggering pyroptosis in various inflammatory diseases, while inhibiting ROS can suppress this process.

AMP-activated protein kinase (AMPK) plays an essential role in the regulation of biological energy metabolism and is implicated in various metabolic diseases (64, 65). It is widely expressed in tissues and organs, activated by diverse stimuli, including cellular stress, exercise, and hormones (64, 66, 67). Recent investigations illustrate that the phosphorylation of AMPK may influence and regulate pyroptosis (68). Zhao Deng et al. demonstrated that the Gly-Pro-Ala (GPA) peptide, isolated from fish skin gelatin hydrolysate, increased the phosphorylation of AMPK and inhibited the pyroptosis process. Additionally, they found that GPA prevented the assembly of the NLRP3 inflammasome and GSDMD cleavage by suppressing ROS (68). Thus, the activation of the AMPK axis suppresses ROS accumulation, blocking NLRP3 inflammasome-mediated pyroptosis (69, 70). In ulcerative colitis, Weiwei Zhang et al. found that the activation of the AMPK/Nrf2 (nuclear factor erythroid 2-related factor 2) signaling pathway, which regulates the antioxidant system, could suppress the NLRP3 inflammasome expression and pyroptosis (71). Thus, these findings suggest that AMPK can be a potential target to regulate the level of ROS and suppress pyroptosis-mediated cell death.

Sirtuins (SIRTs) are a class of evolutionarily highly conserved NAD+-dependent histone deacetylases that regulate key signaling pathways in prokaryotes and eukaryotes and are involved in many biological processes (72). The human SIRT family is composed of seven recognized members, namely SIRT1–7. SIRT proteins can interact with p53, FOXO/PGC-1α, NF-κB, Ku70, and other proteins to regulate processes such as cell stress response, metabolism, aging, and apoptosis (73). Weijie Yan et al. recently found that SIRT1 is upstream of ROS, and ROS bursts lead to increased TNF receptor associated factor 6 (TRAF6) levels. Further, the activation of TRAF6 contributes to pyroptosis. A novel SIRT1-ROS-TRAF6 signaling pathway has been proposed to induce pyroptosis (74). However, other research has indicated that SIRT3 reduces the production of ROS, thereby decreasing the occurrence of pyroptosis (75). This suggests that different SIRTs have distinct functions concerning ROS and pyroptosis.

**Figure 2:**
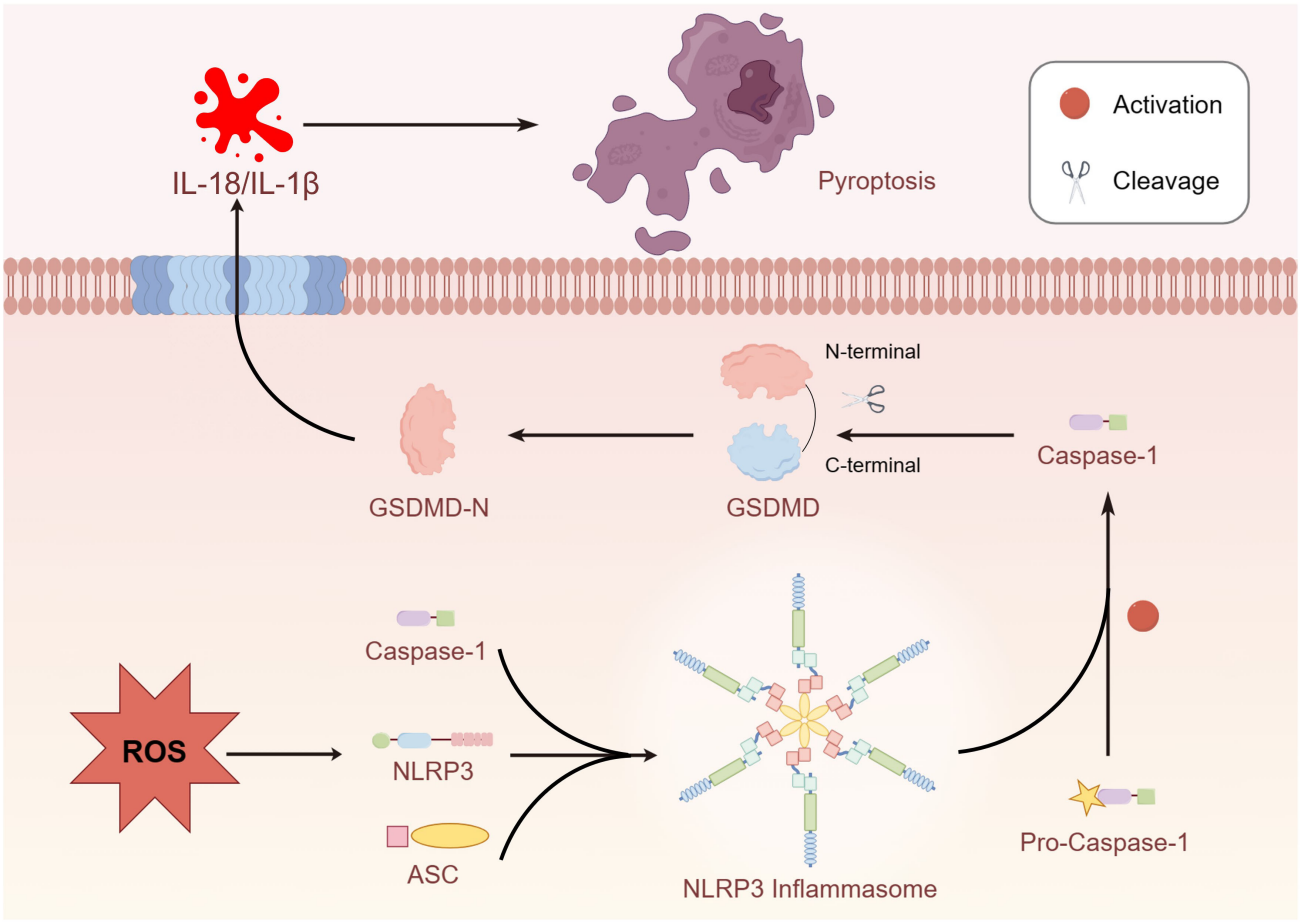
Overproduction of ROS activated NLRP3 and promoted the formation of inflammasome and cleaved the GSDMD due to caspase-1 activation.

Transcription factor E3 (TFE3), a member of the microphthalmia-associated transcription factor (MITF) family, primarily plays a biological role in suppressing ROS production and promoting the expression of lysosome-related proteins (76). However, dysfunction of lysosomal function induced by ROS is a crucial factor in triggering cell pyroptosis (77). Current evidence demonstrates that TFE3 plays a critical role in pyroptosis signaling pathways. Owing to the effect of TFE3 on both ROS and the pyroptosis axis, Jiafeng Li et al. investigated the relationship between these three elements (78). Excessive accumulation of ROS led to lysosomal dysfunction and induced cell pyroptosis, primarily due to the promotion of ROS levels by low expression of TFE3, activating NLRP3-induced cell pyroptosis (77, 79). Additionally, studies have illustrated that the activation of TFE3 is partially mediated by the AMPK signal pathway, preventing lysosomal malfunction induced by the overexpression of ROS (80). **Figure 2**

### 3.2 The “double-edged sword” of ROS regulates pyroptosis in inflammations

Pyroptosis induced by inflammasome activation is an important defense mechanism against bacterial infection, which can protect the host from bacterial infection. The underlying mechanism may involve inflammasome activation, and triggers immune cell pyroptosis defense against pathogen infection via caspase-1/GSDMD pathway (81). In this regard, Edward A Miao et al. reported a protective role of caspase-1 during wild-type (WT) Salmonella systemic infection, potentially due to caspase-1-induced pyroptotic cell death released bacteria from macrophages and exposed the bacteria to uptake and killing by ROS in neutrophils (28). However, excessive pyroptosis can lead to several inflammatory diseases, including sepsis and autoimmune disorders (82). This may be due to the increased expression of NLRP3, apoptosis-associated speck-like protein (ASC), caspase-1, and GSDMD associated with pyroptosis in the host upon infection (83). Notably, in severe bacterial infection, such as those leading to severe inflammatory responses and disseminated intravascular coagulation, bacteria can trigger coagulation disorders through the caspase-11/NLRP3 pathway, ultimately resulting in fatal complications such as septic shock. Studies have shown that the lack of caspase-1 or GSDMD prolonged the survival time in Salmonella infection models and reduced the release of pro-inflammatory cytokines IL-1β, IL-6, and TNFα, and prevented coagulation dysfunction by diminishing prolongation of prothrombin time and increasing plasma thrombin-antithrombin complex concentrations (84). Additionally, the NLRP3 inflammasome plays a critical role in Acinetobacter baumannii infection-induced pneumonia (85), Therefore, inhibiting the activation of the NLRP3 inflammasome has an effective therapeutic effect against inflammatory diseases caused by bacterial infections, which may be achieved by maintaining mitochondrial homeostasis through the suppression of pyroptosis (86). Therefore, in summary, the inflammasome pathway is a double-edged sword in the infectious disease, could be both beneficial and detrimental, depending on the stage of disease and/or the path of infections. While ROS can help kill infectious microorganisms, excessive ROS may cause tissue damage during the inflammatory process, which is closely related to pyroptosis. For example, ROS-mediated caspase-1 activation can exacerbate liver injury during Schistosoma japonicum infection (87), excessive production of ROS can lead to the activation of NLRP3 inflammasome and induce pyroptosis, further exacerbating myocardial damage in viral myocarditis (88).. The primary reason is that in the process of pyroptosis caused by infectious inflammation, excessive ROS disrupt the homeostasis and lead to the development of pathological conditions (89). Therefore, inhibiting the overproduction of ROS, preventing NLRP3 inflammasome activation, and subsequently suppressing the cleavage of GSDMD due to caspase-1 activation, thereby reducing the release of cytokines such as IL-1β and IL-18, can have a protective effect against severe infection-induced fatal inflammatory diseases.

## 4 ROS regulates pyroptosis in cancer

Human tumors consist of diverse malignant and non-malignant cells, forming a complex ecosystem that governs tumor biology and response to treatment (90). Tumors are generally classified into benign and malignant categories (91, 92). ROS are electron-deficient substances (unsaturated electron substances) that compete for electrons throughout the body. If they withdraw electrons from cellular protein molecules, protein alkylation occurs, leading to the formation of distorted molecules and ultimately contributing to carcinogenesis (93–95). Pyroptosis is gasdermin-mediated programmed necrosis that is potentially useful in cancer therapy (96, 97). Therefore, exploring the relationship between ROS and pyroptosis in tumors is of great interest for the treatment and prevention of tumors.

### 4.1 Anti-tumor role of ROS regulated pyroptosis

Currently, studies in tumors have indicated that elevated levels of ROS can induce pyroptosis, which is beneficial in combating cancer. For example, the Nrf2/PPARα molecular axis in breast cancer overcomes chemotherapy resistance and leads to better clinical outcomes by promoting pyroptosis (98). Rongjun Zhang et al. found that nobiletin extracted from citrus fruits can induce cell apoptosis and trigger ROS-mediated pyroptosis by regulating autophagy in ovarian cancer cells (99). In melanoma cells, iron-induced ROS can induce pyroptosis through the Tom20-Bax-caspase-GSDME pathway (49). In cervical cancer, ROS can activate NLRP3 inflammasomes, leading to apoptosis of cervical cancer cells (100). This is possibly because increased levels of ROS induce the assembly of NLRP3 inflammasomes and activation of caspase -1 (101).

Non-small cell lung cancer (NSCLC), the most common type of lung cancer, tends to occur in younger individuals in recent years (102). Fortunately, the activation of ROS-induced pyroptosis has been identified as an inhibitory mechanism against the progression of NSCLC (19, 103). For instance, polyphyllin VI extracted from Trillium tschonoskii Maxim can induce caspase-1-mediated pyroptosis in NSCLC by inducing the ROS/NF-κB/NLRP3/GSDMD signaling axis, providing a possibility of becoming a novel therapeutic agent for NSCLC treatment in the future (19). The PL analogs L50377 can stimulate the production of ROS in NSCLC cells, promoting pyroptosis, and the ROS-mediated NF-κB inhibition may be associated with the mechanism of L50377-induced apoptosis in NSCLC cells (104). Aberrant overexpression of lncRNA-XIST in NSCLC tissues or cells can suppress the development of NSCLC by knockdown of lncRNA-XIST, which activates the miR-335/SOD2/ROS signaling pathway to promote apoptosis (105).

Lobaplatin can enhance the induction of pyroptosis in nasopharyngeal carcinoma cells through the generation of ROS (106). In colon cancer cells, GSDME mediates Lobaplatin-induced ROS/JNK/Bax-mitochondria apoptosis pathway and pyroptosis activation downstream of caspase-3/-9 (107). Overexpression of Thioredoxin Reductase 3 (Txnrd3) may increase ROS production by inducing intracellular calcium efflux, followed by activation of the pyroptosis pathway, further inhibiting the growth and proliferation of colon cancer cells (108). Coxsackievirus B3 (CVB3) causes GSDME cleavage by activating caspase-3, ROS can also promote CVB3-induced pyroptosis, and CVB3 exhibits oncolytic activity in colon cancer cell lines through GSDME-mediated pyroptosis (109). Simvastatin possesses anti-tumor effects by downregulating ROS production and inducing downstream caspase-1-dependent pyroptosis. Specifically, simvastatin induces pyroptosis through the ROS/caspase-1/GSDMD pathway and serves as a potential drug for the treatment of colon cancer (110).Mammalian STE20-like kinase 1 (MST1) also partially inhibits the progression of pancreatic ductal adenocarcinoma (PDAC) cells through ROS-induced pyroptosis (111).

Some novel technologies have shown promising results in demonstrating the benefits of ROS and pyroptosis in tumors. For example, cold atmospheric plasma (CAP) can effectively induce pyroptosis in tumor cell lines with high expression of GSDME in a dose-dependent manner by inducing ROS (112). Hydrogen is also likely to alleviate tumor volume and weight in the xenograft mouse model through the ROS/NLRP3/caspase-1/GSDMD-mediated pyroptotic pathway (113). The use of nanosystems camouflaged with homogeneous cell membranes composed of hybrid membranes and corresponding mitochondrial membranes provides a complex drug (Ca@GOx) composed of calcium phosphate and glucose oxidase (GOx), which utilizes the homing effect of cell membranes and the directed fusion mechanism of subcellular membranes to deliver Ca@GOx to mitochondria. This leads to mitochondrial Ca^2+^ overload, generating significant levels of ROS, resulting in pyroptosis, and enhancing the tumor’s immunogenic response (114). The lack of tumor antigens results in a low response rate, which is a major challenge in immune checkpoint blockade (ICB) therapy. A nanotherapeutic predrug strategy that stimulates photodynamic chemotherapy may be a smart approach to trigger pyroptosis and increase the efficiency of ICB (115). Iron-containing metal-organic framework materials (TPL@TFBF) functionalized with BSAFA induce immunogenicity by inducing pyroptosis in tumor cells and releasing large amounts of damage-associated molecular patterns (DAMPs), showing remarkable efficacy against melanoma lung metastasis *in vivo*, thereby eliciting an effective systemic antitumor immunologic response. This is mainly due to the amplification of intracellular ROS, which induces cell pyroptosis (116).

### 4.2 Pro-tumor role of ROS regulated pyroptosis

In addition, in the tumor microenvironment, the interaction between inflammatory cell infiltration and stromal cells is intricate, inflammation not only exerts anti-tumor effects but also promotes tumorigenesis (117). This is inseparable from the duality of ROS, where low levels of ROS promote tumors, while excessive ROS induce various forms of tumor cell death (118). Pyroptosis plays a non-negligible role in inflammation promoting tumor development, particularly linked to inflammasome-induced chronic inflammation, and it has been demonstrated that tumor initiation is influenced by chronic inflammation (119, 120). The main reason may lie in the ability of ROS to activate the NLRP3 inflammasome, promoting the invasion and migration of tumor cells (121). For example, bile acids (BA) activate the inflammasome by causing excessive accumulation of ROS, ultimately inducing pyroptosis, promoting the development of hepatocellular carcinoma (HCC) (122). Additionally, periodontitis (a type of chronic inflammation) promotes breast cancer metastasis in the early stages through pyroptosis (123), Zunjie Zhou et al. discovered that pyroptosis may promote glioblastoma development by inducing chronic inflammatory microenvironments (124), indirectly proving the role of ROS-induced chronic inflammation in promoting tumors through pyroptosis.

## 5 ROS regulates pyroptosis in other diseases

Autophagy is closely associated with cerebral ischemia/reperfusion injury, wherein cerebral ischemia/reperfusion leads to increased ROS levels and upregulation of autophagy- and pyroptosis-related proteins (125). Additionally, pre-treatment with spautin-1 reduces autophagy and ROS accumulation and attenuates NLRP3 inflammasome-dependent pyroptosis, suggesting that inhibiting autophagy may be a promising approach for treating cerebral ischemia/reperfusion injury (126). Ti2C@BSA-ISO nanocomposite, wrapping isoquercitrin (ISO) on Ti2C nanoenzyme, serves as a novel therapeutic nanomedicine targeting ROS for the treatment of ischemic stroke. Specifically, Ti2C@BSA-ISO alleviates ischemic stroke by inhibiting the NLRP3/caspase-1/GSDMD pathway-mediated pyroptosis (127). Furthermore, bilirubin (Br) and atorvastatin (As) are encapsulated into an intelligent ROS-responsive nanocarrier DSPE-TK-PEG (DPTP) to form nanoparticles (BA@DPTP), which effectively restrain the NF-κB signaling transduction and NLRP3/caspase-1/GSDMD-dependent pyroptosis in mouse pulmonary microvascular endothelial cells, reducing the mortality associated with acute lung injury (128).

Reticulocalbin-2 (RCN2) is involved in regulating vascular inflammation and plays a vital role in the cardiovascular system (63). Jun Kai et al. recently found that RCN2 knockdown significantly reduces pyroptosis, the release of lactate dehydrogenase (LDH) and IL-1β, and ROS production and inhibits the expression of pyroptosis-related proteins (NLRP3, cleaved caspase-1, and cleaved GSDMD) (63). Diabetic nephropathy (DN) is a severe complication of diabetes, with limited treatment options, but the role of pyroptosis in the pathogenesis of DN is of great significance (129, 130). Guangru Li et al. showed that syringaresinol (SYR) ameliorated renal hypertrophy, fibrosis, mesangial expansion, glomerular basement membrane thickening, and podocyte foot process effacement in streptozotocin (STZ)-induced diabetic mice by upregulating the Nrf2 signaling pathway to inhibit the NLRP3/caspase-1/GSDMD pyroptosis pathway (131).

In addition, organelles are critically involved in the process of pyroptosis induced by ROS (132). The endoplasmic reticulum (ER), an important organelle involved in protein synthesis, storage, folding, processing, modification, assembly, and transport, is central to cell function (133). External stimulus causing incomplete or incorrect protein folding in the ER leads to ER stress (134). Continuous or strong ER stress activates signaling pathways that result in cell damage or death (135). Recent reports indicate that ER stress is closely related to cell pyroptosis. For example, Xiaohan Yang et al. found that in the process of renal ischemia/reperfusion injury in mice, the expression of C/EBP-homologous protein (CHOP) was significantly increased. The expression of caspase-11 mRNA mediated the induction of pyroptosis (136). Kinsella Sinéad et al. demonstrated that elevated mitochondrial ROS levels promote pyroptosis in thymocytes after acute insult by driving caspase 1 cleavage. They showed that pyruvate drives this increase in mitochondrial ROS, further triggering caspase-1 cleavage and pyroptosis (137).

## 6 Compounds that affect ROS and induce pyroptosis

Currently, in inflammatory diseases, various compounds, including eugenol, oroxylin, pioglitazone, trimethylamine N-oxide, and berberine, have been identified for inhibiting pyroptosis by influencing ROS-related pathways, as outlined in **Table 1**. These compounds possess strong electron-donating groups, such as -OH and -NH, or strong electron-withdrawing groups, such as N^+^. They can activate or inhibit ROS, similar to the excessive ROS levels in cells, thereby activating relevant signaling pathways. In tumor diseases, several compounds, such as lobaplatin, L50377, and nobiletin, have been found to influence ROS and trigger pyroptosis for cancer cell eradication (**Table 1**).

**Table 1:** Compounds induce pyroptosis through ROS.

Name	Structure	Pathway	References
Eugenol	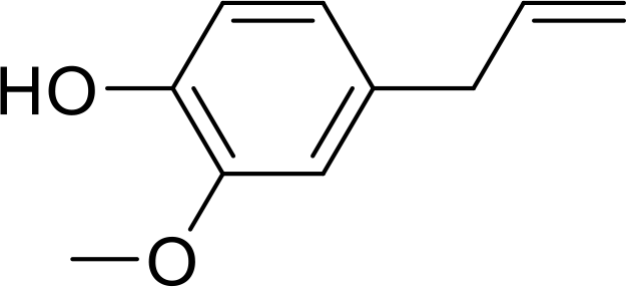	ROS/NLRP3/GSDMD	(138)
Oroxylin	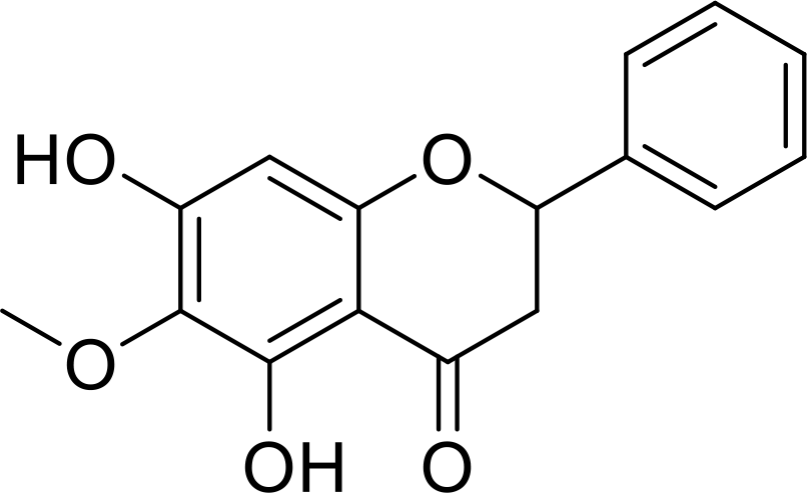	PGC-1α/Mfn2/ROS	(62)
Pioglitazone	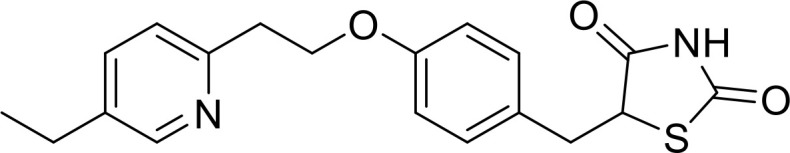	Rac1/ROS/PPAR-&	(139)
Trimethylamine N‐oxide	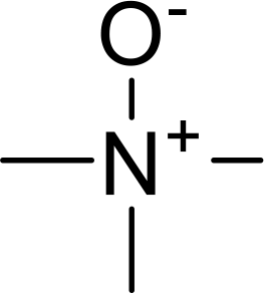	SDHB/ROS	(140)
Berberine	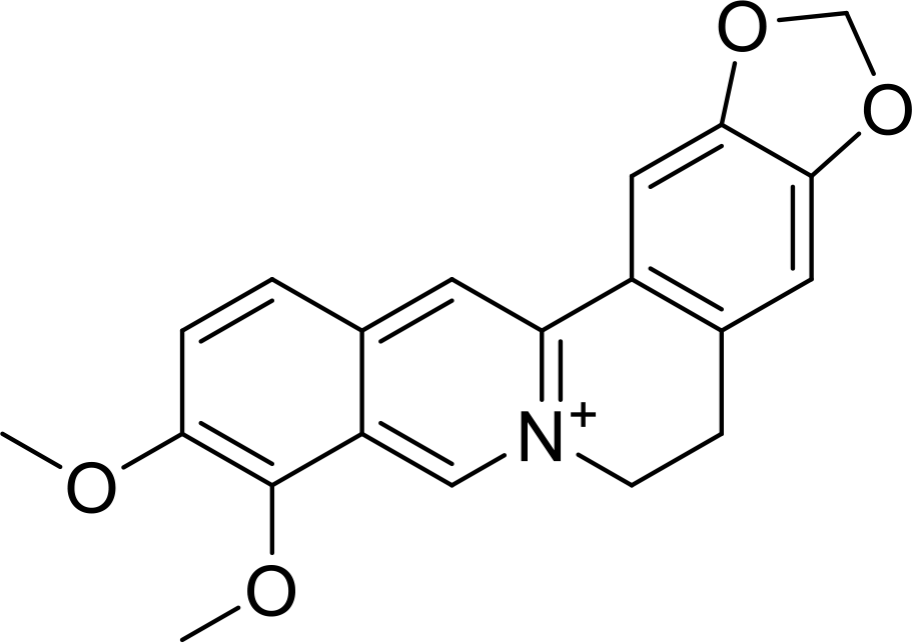	ROS/TXNIP	(141)
Lobaplatin	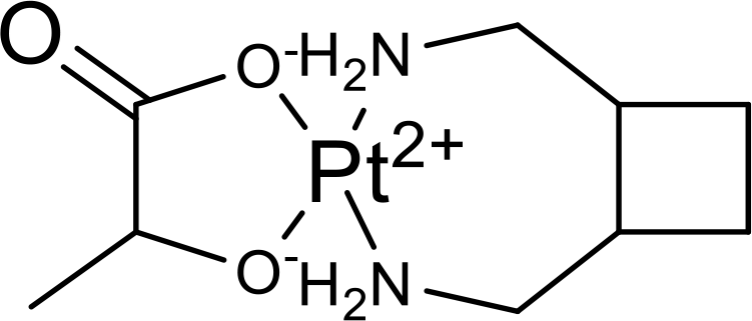	cIAP_1/2_/Ripoptosome/ROS	(106)
L50377	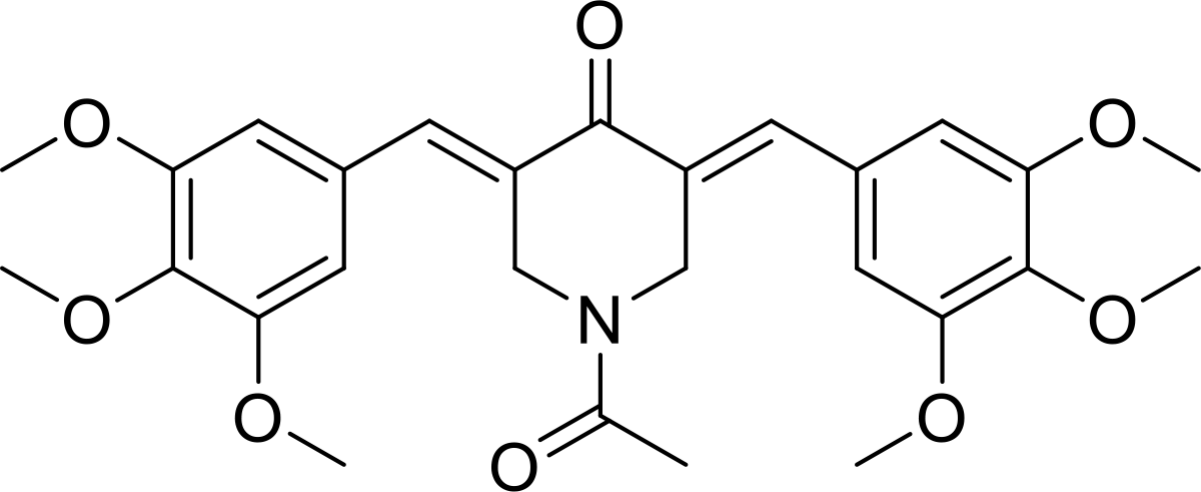	ROS/NF-κB	(104)
Nobiletin	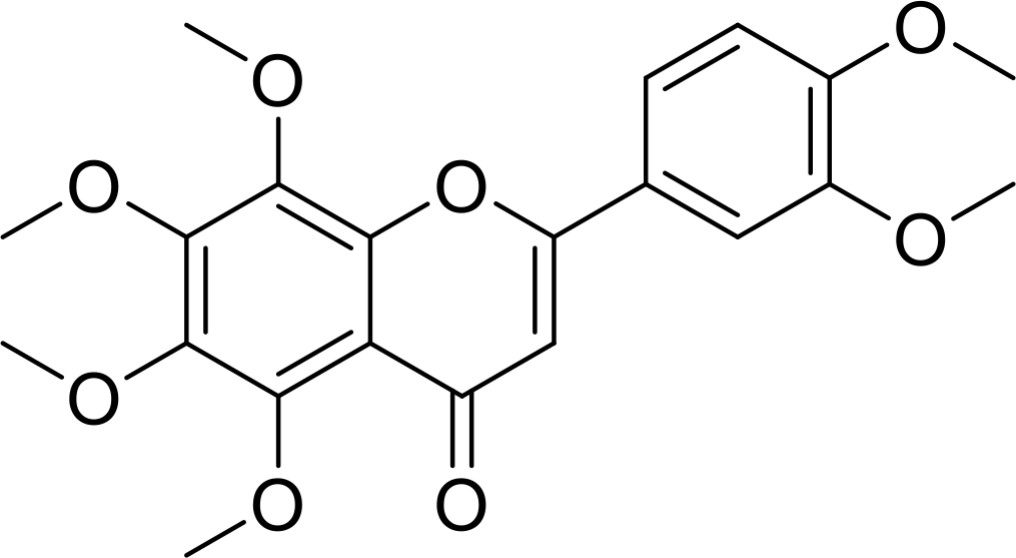	ROS	(99)
Melatonin	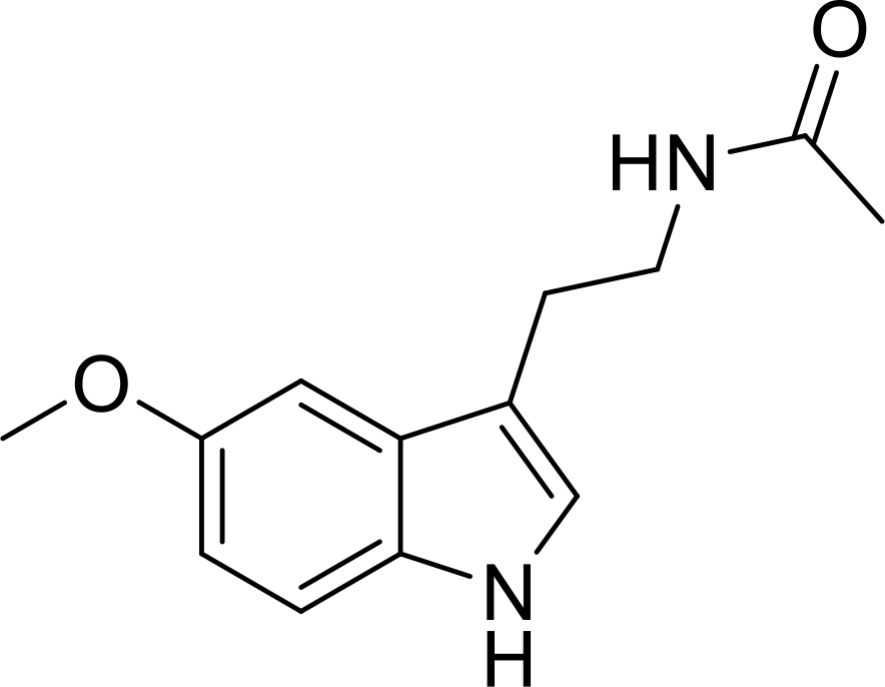	ROS/NLRP3	(60)
α-KG	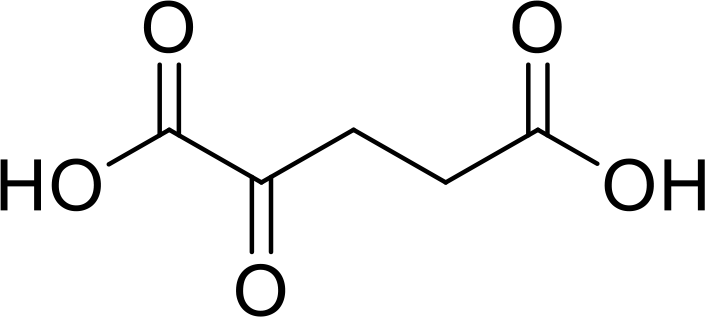	ROS/DR6/GSDMC	(53)
5-Fu	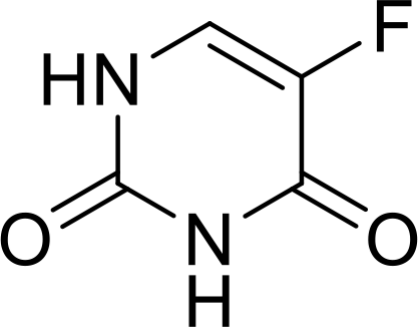	ROS/NLRP3	(142)
Chalcone derivative 8	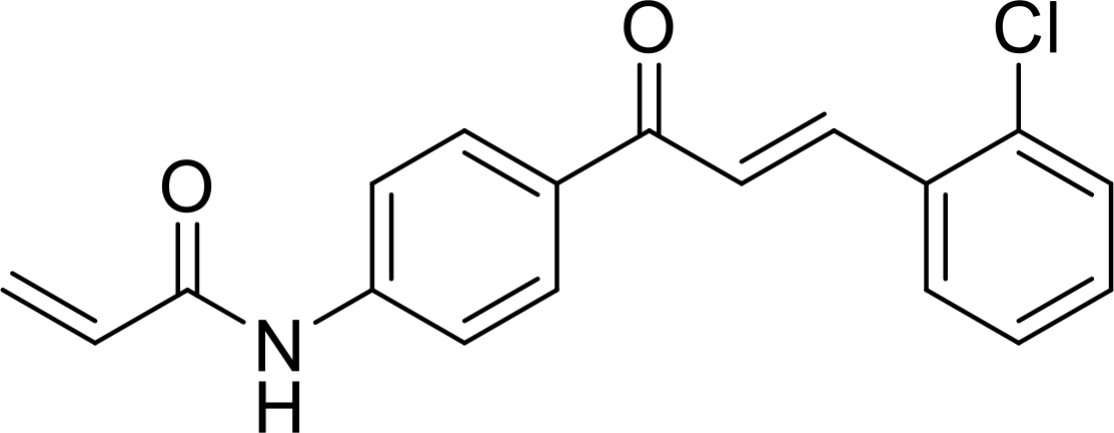	ROS/caspase-3	(143)
Polyphyllin VI	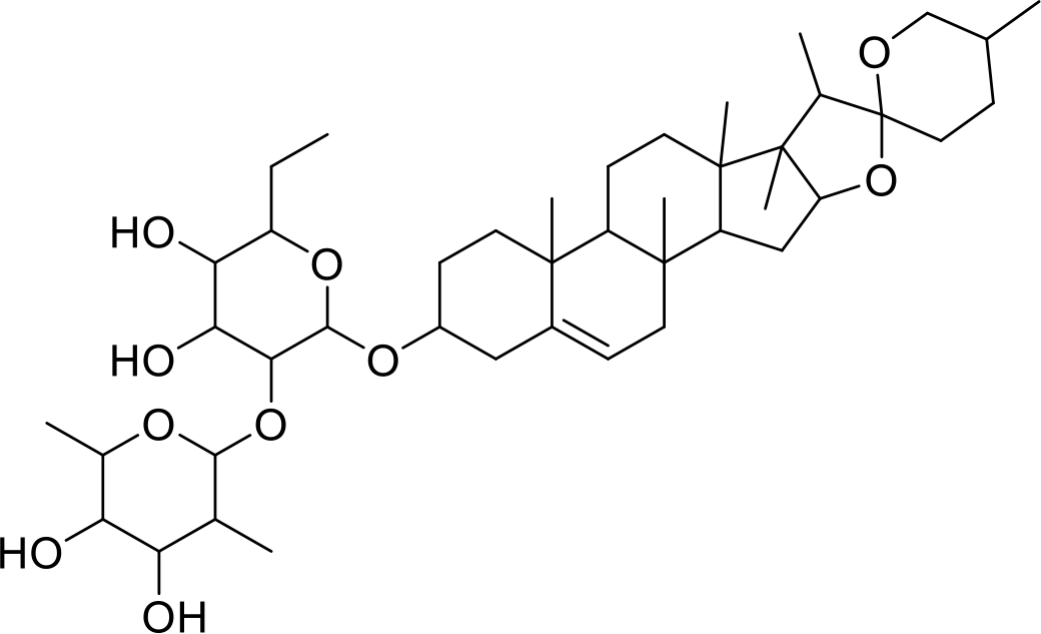	ROS/NF-κB/NLRP3/caspase−1/GSDMD	(19)
Simvastatin	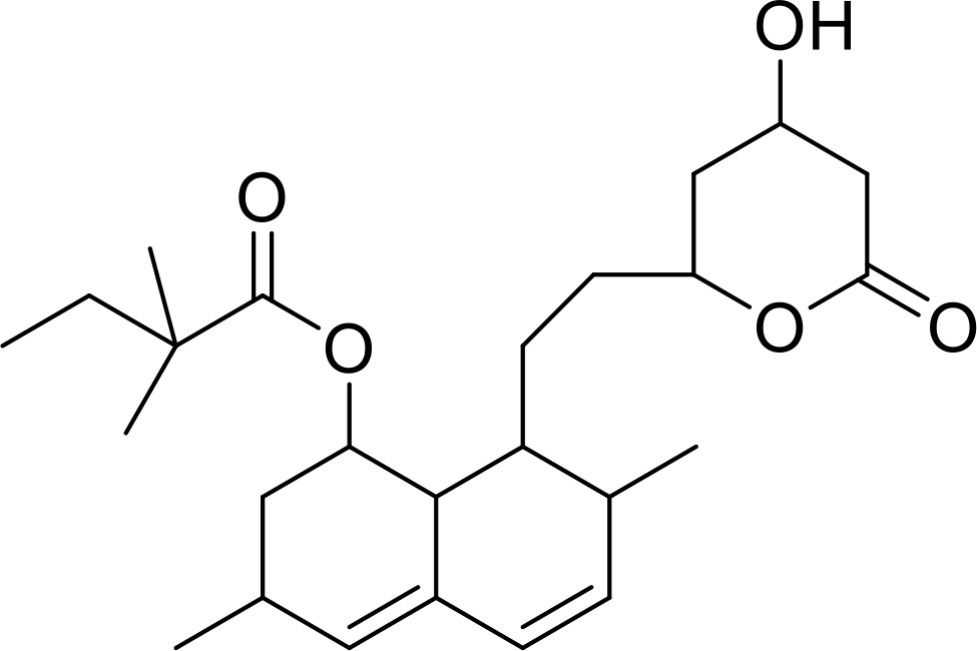	ROS/caspase-1/GSDMD	(110)

Lobaplatin, a frontline antitumor drug with excellent anticancer activity, is primarily used for treating ovarian cancer, NSCLC, esophageal cancer, gastric cancer, and other cancers. Zide et al. discovered that lobaplatin induces pyroptosis in nasopharyngeal carcinoma by regulating ROS through cLAP1/2. L50377, as flavonoid compounds, directly regulate ROS to induce pyroptosis in cancer cells. Simvastatin was mainly used in the treatment of hyperlipidemia in the past, but recently it has been rediscovered for exhibiting anticancer activity. Recently, it was indicted that simvastatin induced HCT116 and SW620 cell pyroptosis and suppressed cell proliferation by down-regulating ROS production and inducing downstream caspase-1. Nobiletin, a prospective food-derived phytochemical extracted from citrus fruits, has recently been reported to suppress ovarian cancer cells. Rongjun Zhang et al. found that nobiletin could significantly inhibit cell proliferation, induce reactive oxygen species (ROS) generation and autophagy of ovarian cancer cells, contributing to gasdermin D-/gasdermin E-mediated pyroptosis. Chalcone derivative 8, bearing two α, β−unsaturated ketones cause intracellular ROS accumulation, thereby triggering caspase−3−mediated pyroptosis in lung cancer cells, which displayed anticancer efficacy and good safety profile. Furthermore, study found that Polyphyllin VI (PPVI) promotes pyroptosis by activating the ROS/NFκB/NLRP3/caspase 1/GSDMD signal axis, which also suppressed the proliferation of NSCLC.

As a common antioxidant, xuebin wang et al. found that melatonin could attenuate cigarette smoke (CS)-induced endothelial cell (EC) pyroptosis by inhibiting ROS/NLRP3 axis. Xiaodong Feng et al. also indicated that antioxidant N-acetylcysteine (NAC) targets ROS/NLRP3 the inflammasome/IL-1β signaling pathway may be helpful for 5-FU adjuvant chemotherapy for OSCC. It was known that cell fate was closely related to metabolic homeostasis in cells, for example α-ketoglutarate (α-KG) as a chemical factor affected pyroptosis. α-KG was converted by the metabolic enzyme MDH1 into another metabolite (L-2-hydroxyglutarate), which results in increased ROS levels and the N-terminal of GSDMC perforates the cell membrane.

These compounds contain olefine ketone groups, which likely play a major role as functional groups. However, to date, no definitive conclusions exist regarding compounds that can induce or inhibit pyroptosis. Designing and studying the functional groups of these compounds hold profound significance for understanding the onset and progression of inflammatory or tumor diseases.

## 7 Conclusion and prospect

Pyroptosis, also known as inflammatory programmed cell death, is induced by the activation of the caspase family and cleavage and polymerization of gasdermin family members, leading to cell membrane rupture and cell death. ROS play a crucial role in signal transduction, regulating multiple signaling pathways, including immune-inflammatory factors, and hold an irreplaceable position in various biological processes. However, systematic research on the effect of ROS on pyroptosis in tumors and inflammation, along with its mechanisms, is currently lacking. Thus, in this study, we collected relevant literature to summarize and elucidate the effects and mechanisms of ROS on pyroptosis in both inflammatory and tumor contexts.

**Figure 3:**
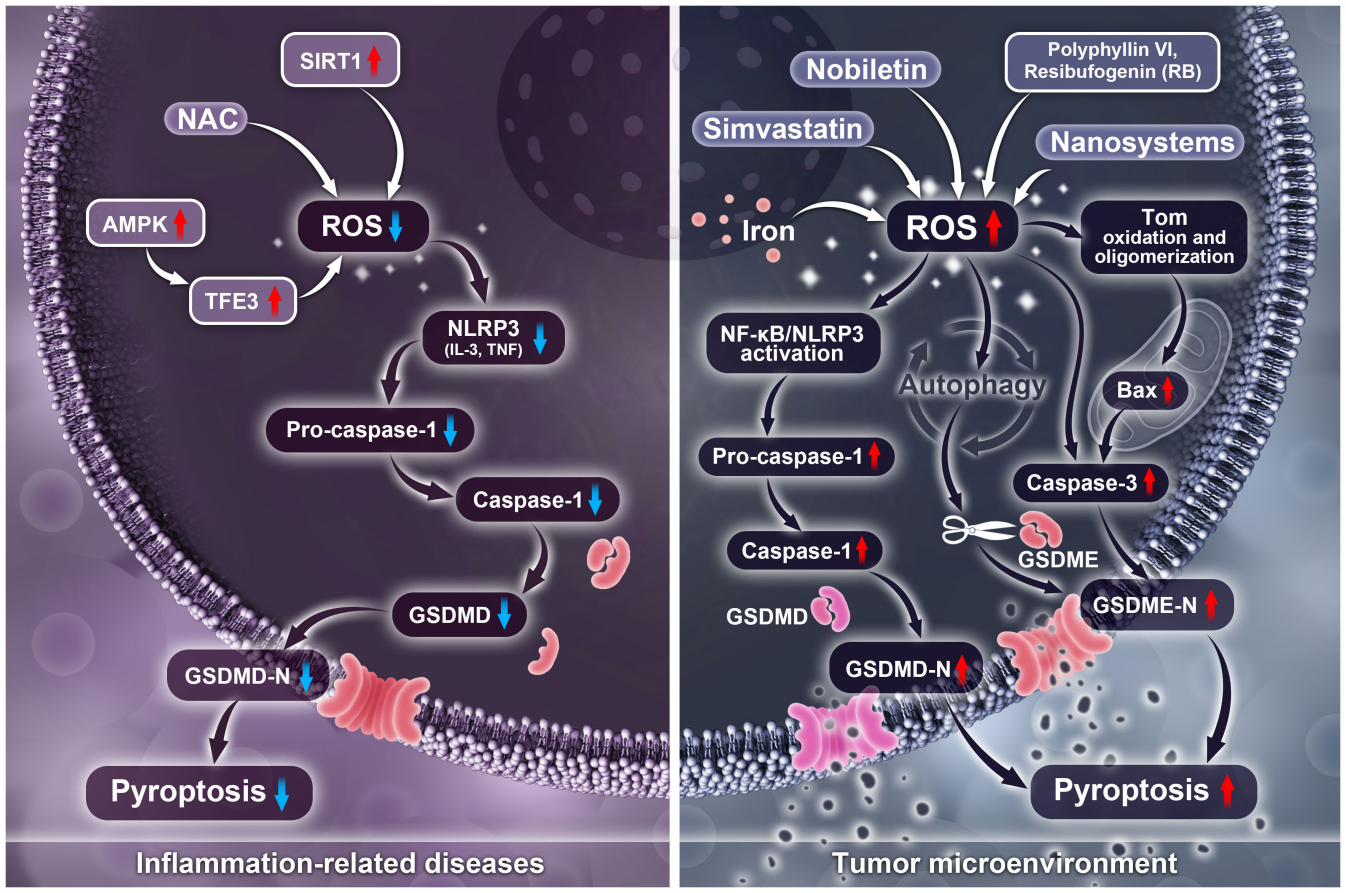
Differences of ROS regulates pyroptosis in tumor and inflammatory diseases.

In inflammation-related diseases, ROS-induced pyroptosis is detrimental, and inhibiting ROS can suppress the activation of the NLRP3 inflammasome, subsequently inhibiting the cleavage of GSDMD to suppress pyroptosis. Currently, several approaches inhibit pyroptosis by targeting ROS, including treatment with the antioxidant NAC, activation of the SIRT1 protein, inhibition of ER stress, promotion of GPA synthesis to induce p-AMPK, or regulation of TFE3 through AMPK. However, ROS plays the opposite role in tumors. Activation of ROS can induce inflammation or activate caspase-3, resulting in the cleavage of GSDMD/E and triggering pyroptosis in tumor cells, thereby exhibiting anti-tumor effects. Particularly, polyphyllin VI, RB, and hydrogen activate ROS to induce NLRP3/NF-κB activation and the cleavage of GSDMD, which is triggered by inflammation. Nobiletin activates ROS to induce cell autophagy, while iron and CAP activate ROS to promote apoptosis-related protein caspase-3 activation, subsequently triggering the cleavage of GSDME and leading to pyroptosis (**Figure 3**). Additionally, in diseases such as stroke and DN, inhibiting ROS-induced pyroptosis exhibits protective effects. It is worth noting that low levels of ROS play a protective role in inflammation caused by bacterial infections, while mild chronic inflammation induced by small amounts of ROS can also promote tumor immune escape through pyroptosis.

This review summarizes the major roles of ROS and pyroptosis and their complex interplay in diseases, indicating that the interplay between ROS and pyroptosis may serve as a potential therapeutic target for diseases such as tumors. Furthermore, we acknowledge that key proteins associated with ROS and pyroptosis, such as NLRP3, caspase-1, GSDMD, and GSDME, may be potential targets for future therapeutic drugs. Some nanomaterials may serve as novel therapeutic approaches targeting ROS and pyroptosis. However, considering the differences in ROS and pyroptosis in different disease contexts is essential. To validate these preliminary findings and evaluate the value of the association between ROS and pyroptosis, more intensive *in vitro* and *in vivo* studies are necessary.

## Author contributions

JW: Writing – review & editing, Writing – original draft. MZ: Writing – original draft. YZ: Writing – original draft. JX: Writing – review & editing, Writing – original draft. ZW: Writing – review & editing.
